# Serpiginous erythema multiforme

**DOI:** 10.1093/omcr/omag122

**Published:** 2026-07-12

**Authors:** Jamie D P Wen, Dimitrios Karponis

**Affiliations:** University College London Medical School – Faculty of Medical Sciences, 74 Huntley Street, London, WC1E 6BT, United Kingdom; University College London Medical School – Faculty of Medical Sciences, 74 Huntley Street, London, WC1E 6BT, United Kingdom; Norwich Medical School, University of East Anglia, Norwich Research Park, Norwich, NR4 7JT, United Kingdom

**Keywords:** erythema multiforme, serpiginous rash

A 21-year-old female presented with a 3 week history of a painful and pruritic rash on her left thigh, subsequently involving the buttocks and right thigh. She reported no preceding illness, unwell contacts, recent travel, allergies, new or regular medication use. There was no relevant family history. Prior empirical treatment with oral flucloxacillin in primary care offered no benefit.

**Figure 1:**
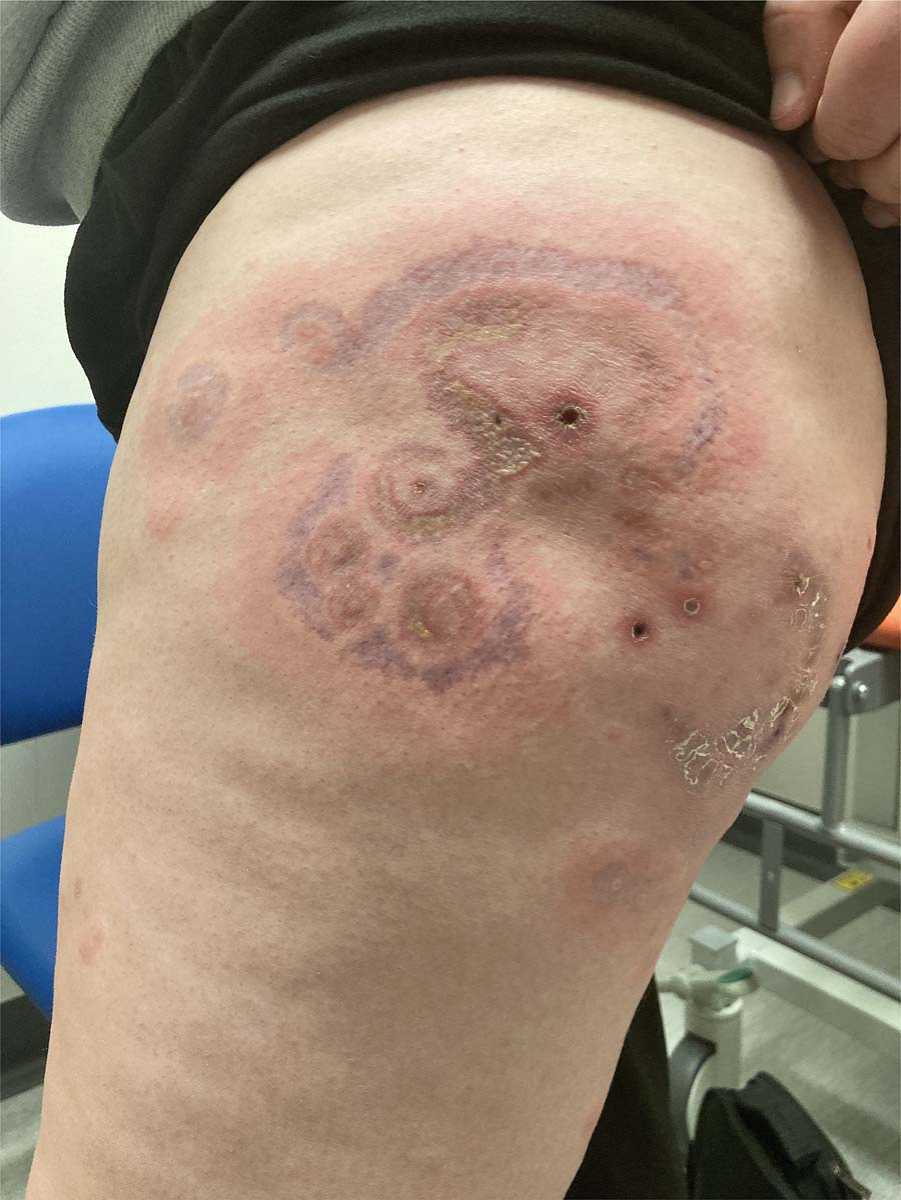
Left lateral thigh showing serpiginous, violaceous streaks with surrounding indurated erythema. Two vesicles are visible on the satellite lesion on the posterolateral thigh. Reproduced with consent.

Examination revealed serpiginous, violaceous streaks with surrounding indurated erythema. Two vesicles were identified within the lesions (Fig. 1). There was no mucosal involvement. The differential diagnosis included erythema multiforme (EM), urticarial vasculitis and erythema gyratum repens.

Laboratory investigations showed normal full blood count, renal profile, liver function tests and C reactive protein. Vesicular bacterial and viral swabs (including for Herpes Simplex Virus types 1 and 2), along with throat viral polymerase chain reaction were negative. An incisional biopsy from the left thigh showed coagulative epidermal necrosis, intraepidermal bulla with mixed inflammatory infiltrate and a dense perivascular and periadnexal lymphohistiocytic infiltrate extending to the subcutis. There were no histological features, other organ involvement or symptoms to suggest cutaneous or systemic vasculitis. The patient did not have a background of malignancy, and her rash did not progress rapidly or concentrically to support erythema gyratum repens.

A diagnosis of EM was made. Despite poor treatment adherence to clobetasol propionate 0.05% ointment, prescribed for symptomatic relief, lesions resolved spontaneously, leaving post inflammatory hyperpigmentation at 2-month follow-up.

EM is more common in men and young adults, with an average onset between 20 and 40 years [1]. Causes include infections (most commonly *Herpes Simplex Viruses* or *Mycoplasma pneumoniae*), medications, autoimmune disease, malignancy, immunisations, contact dermatitis and menstruation [2]. Treatment is supportive in the absence of a reversible trigger, which may be unidentified in up to 60% of cases [3].

This case highlights an unusual presentation of histologically-confirmed idiopathic EM with serpiginous indurated streaks.
